# Effects of Processing Methods and Conditioning Temperatures on the Cassava Starch Digestibility and Growth Performance of Broilers

**DOI:** 10.3390/ani13081373

**Published:** 2023-04-17

**Authors:** Xuan Wang, Baolong Du, Fang Nian, Yingjun Ru, Likun Sun, Shizhen Qin, Defu Tang

**Affiliations:** 1 College of Animal Science and Technology, Gansu Agricultural University, Lanzhou 730070, China; 2 Diasham Resources Pte. Ltd., Singapore 629314, Singapore

**Keywords:** mechanical crushing, steam conditioning, puffing conditioning, starch digestibility

## Abstract

**Simple Summary:**

The rapid development of the cassava industry has increased our demand for dried cassava, but the supply of raw materials and the production of cassava starch have declined due to the reduction in the planting area. Moreover, the anti-nutritional factors contained in natural cassava limit the application of cassava. Therefore, it is necessary to study processing methods to improve the utilization value of cassava. We used three processing methods (mechanical crushing, steam conditioning, and puffing conditioning) and three conditioning temperatures (60, 75, and 90 °C) to process cassava and cassava feed so as to improve the utilization of cassava. The results found that high-temperature conditioning reduced broilers’ growth performance and nutrient utilization, and steam conditioning and puffing conditioning increased the in vitro starch digestibility and nutrient utilization of broilers. Specifically, cassava starch had lower amylose content and amylose/amylopectin at a conditioning temperature of 60 °C in combination with puffing conditioning or steam conditioning, and puffing conditioning had the highest crude protein digestibility (in the starter period) and apparent metabolizable energy (in the grower period) at 60 °C.

**Abstract:**

As an important food crop, cassava is rich in nutrients and high in starch content and is widely used in the production of industrial raw materials. However, the utilization value of cassava is limited due to the reduction of planting area and the existence of anti-nutritional factors. Therefore, we evaluated in vitro cassava starch digestibility and in vivo growth performance of broilers in a 3 × 3 factorial arrangement of treatments using three processing methods (mechanical crushing (MC), steam conditioning (SC), and puffing conditioning (PU)) and three conditioning temperatures (60, 75, and 90 °C) to screen for the optimal processing method and conditioning temperature to improve the utilization of cassava. In the in vitro cassava starch digestion study, the digestibility and digestion rate (*p* < 0.01) were higher at conditioned 90 °C than that at 60 or 75 °C, and PU was higher than SC and MC (*p* < 0.01) (0.25–2 h). The amylose content and amylose/amylopectin at conditioned 60 °C or PU were lower (*p* < 0.01) than that of 75 or 90 °C or SC, whereas the opposite was true for amylopectin content (*p* < 0.01). The resistant starch content of SC or PU was lower (*p* < 0.01) than MC. In the in vivo study, broilers fed diets conditioned at 60 °C or SC had a lower (*p* < 0.05) feed-to-gain ratio than those fed diets conditioned at 90 °C or PU diets. The ileum apparent digestibility of starch and AME were higher (*p* < 0.05) for broilers fed SC diets than for those fed MC diets. These results indicate that cassava starch promoted starch digestion rate by reducing amylose content and amylose/amylose under PU combined with a conditioning temperature of 60 °C, ileum digestibility of starch in broilers fed SC diets was higher than MC diets regardless of conditioning temperature, and SC diets increased AME and decreased F/G to promote growth performance of broilers.

## 1. Introduction

Cassava (*Euphorbiaceae* family), sweet potato, and potato are known as the world’s three major potato crops, of which cassava is highly adaptable, resistant to drought and acidic soil, and can be grown on poor land. At the same time, cassava is a potentially high biomass-producing crop [1]. Widely planted in tropical and some subtropical countries and regions [2,3]. In recent years, with the continuous development of the cassava industry and the gradual diversification of uses [2,4], the demand for dried cassava in China has increased significantly, reaching 7.1827 million tons in 2021, a year-on-year increase of 43.9% [5]. However, the cassava planting area continues to shrink, which leads to a continuous reduction in domestic raw material supply and a significant reduction in domestic cassava starch production. In 2021, China’s cassava starch production was 211,800 tons, a year-on-year decrease of 19.23% [5]. In addition, native cassava contains anti-nutritional factors (hydrocyanic acid, tannin) that reduce animal intake and the digestion and absorption of nutrients (such as protein, etc.), limiting the use of cassava in animal husbandry [3]. In the feed industry, methods such as mechanical crushing (MC), steam conditioning (SC), puffing conditioning (PU), or conditioning temperature are often used to process feed raw materials, which can not only change the nutritional value of the feed but also change the digestion rate and digestibility of starch, thereby improving its utilization value.

The starch content in feed has always been a concern of animal husbandry scientists. The starch content in fresh cassava roots is as high as 32%, amylose generally accounts for 20–30% of the total starch content, and amylopectin accounts for 70–80% of the total starch content [6]. Amylopectin has a larger surface area than amylose and can bind to more amylase, while the glucose chains of amylose are mostly bound together by hydrogen bonds, which makes amylose more difficult to be hydrolyzed by amylase than branched starch [7]. The rate and site of starch digestion in vivo depend on the content of amylose and amylopectin [8]. Cassava starch is more quickly digested because it contains less amylose than most grains, legumes, and potato starches [9]. Generally, starch be classified as rapidly digestible starch (RDS), slowly digestible starch (SDS), and resistant starch (RS) according to the digestion time of starch in the small intestine [10]. The rate and extent of starch digestion regulate the postprandial metabolism of broilers. The RDS releases glucose at a faster rate at the front of the small intestine, leading to a decrease in the energy supply of glucose in the jejunum and ileum, an increase in fecal nitrogen excretion, and a decrease in ileum nitrogen retention, thereby improving the digestion and absorption function of animals [11].

Different processing methods and conditioning temperatures vary and affect the extent and rate of starch digestion, which may influence the balance between nutritional effectiveness and diet quality [12,13]. MC is the first step in feed production, mainly through physical effects such as friction, collision, and shear force to change the structure as well as physicochemical properties of starch granules, thereby affecting the digestibility of starch [14,15,16]. The crushing disrupts the crystalline regions of starch and increases the effective contact between digestive enzymes and starch granules to promote the digestion rate of starch [16,17]. The SC induces the starch to absorb water and expands to a gelatinization state during heating [12]. By breaking the hydrogen bonds in starch granules, starch molecules become single molecules, protein denaturation, starch gelatinization, or anti-nutritional factors such as trypsin inhibitors were inactivated to improve starch digestibility [18,19]. The puffing conditioning destroys the crystalline structure of native starch [20], reduces the crystallinity of starch, and increases the gelatinization temperature, which has better thermal stability [21]. Changes in starch internal structure and starch granule size caused by puffing conditioning increased starch digestion rate and starch digestibility [22]. Compared with MC, PU increased the digestion rate of feed starch by increasing the content of RDS in grains and obtained a higher starch digestion coefficient [23]. The SC and PU destroyed the endosperm cell wall of the feed material and the intermolecular hydrogen bond that maintained the starch crystal structure and improved the apparent digestibility of starch in the ileum and feed conversion rate of animals [24,25]. Appropriate conditioning temperature not only effectively kills potential pathogens in feed materials [26] but also improves starch digestibility and the digestion and utilization of nutrients in feed by animals by destroying the crystal structure and gelatinization degree of native starch [27,28].

Based on the fact that SC, PU, and appropriate conditioning temperature could alter starch digestibility and had a positive effect on animal growth, we hypothesized that the combined application of processing methods and conditioning temperatures would increase the digestibility of cassava starch in vitro and improve the broiler growth performance. Therefore, the purpose of this study is to analyze the effects of different processing methods and conditioning temperatures and their interaction on the digestibility of cassava starch, the growth performance of broilers, and the digestibility and utilization of nutrients.

## 2. Materials and Methods

This study protocol was reviewed and approved by the Animal Welfare Ethics Committee of Gansu Agricultural University (Approval No. DK-019). The animal procedures used in this study strictly abide by the Administrative Measures of Gansu Province on Experimental Animals (2021–2025). Glucose and starch standard samples were purchased from Shanghai Zhanyun Chemical Co., Ltd. (Shanghai, China). All other reagents used in this study were analytical grade and were purchased from Sinopharm Chemical Reagent Co., Ltd. (Shanghai, China).

### 2.1. In Vitro Study and Design

#### 2.1.1. Preparation of Cassava

The study was a completely randomized design with 3 processing methods (mechanical crushing (MC), steam conditioning (SC), and puffing conditioning (PU)) × 3 conditioning temperatures (60, 75, and 90 °C). The cassava roots were cut into small pieces using a slicer machine (YC-75, Guangzhou Jinben Machinery Equipment Co., Ltd., Guangzhou, China) after removing peels, stems, leaves, purified and impurities, then sun-dried on a concrete floor for about 2–3 days until the moisture content dropped below 14% [29], then ground with a hammer mill (CM 100, Beijing Grinder Instrument Co., Ltd., Beijing, China). The cassava obtained by MC was conditioned at 85 °C using a steam regulator (YSCD, Shanghai Yusheng Machineries Co., Ltd., Shanghai, China) at 2.5 kPa for 30 s to obtain SC cassava. The PU (dry puffing) cassava was obtained using an expanding machine (YSEX, Shanghai Yusheng Machineries Co., Ltd., Shanghai, China) for 5 s at 130 °C. Other cassava was conditioned for 30 s at 60, 75, or 90 °C with a pelletizer (SZLH420, Zhongmu Feed Machinery Co., Ltd., Liyang, China) equipped with a ring dies with a die hole diameter of 3 mm and a thickness of 35 mm. The conditioning temperature was based on the temperature at the outlet of the modulator by a thermal probe. The hydrocyanic acid content of cassava was 15.0~20.0 mg·kg^−1^.

#### 2.1.2. Extraction of Cassava Starch

The extraction of cassava starch was conducted as described by El Halal et al. (2019) [30]. Cassava samples were placed in distilled water (*v*/*w* = 10:1) overnight. The mixture was ground in a mortar and filtered through 200 mesh sieves. The filtrate was then centrifuged at 3000× *g* for 15 min. The upper layer containing impurities was removed. The precipitate was suspended in distilled water and then centrifuged again. This process was repeated several times until the supernatant was clear. The sediment was dehydrated with absolute ethanol twice, dried at 40 °C for 6 h, then ground into powder, screened through 100 mesh sieves, and stored at −20 °C. The determination of starch content refers to GB 5009.9-2016, and the content of amylose refers to GB/T 15683-2008.

#### 2.1.3. In Vitro Digestibility of Cassava Starch

The in vitro digestibility property of cassava starch was determined based on a previously published method that simulated the digestive behavior of the digestive tract of the broiler [31] with modifications according to Englyst et al. [10]. Three replications of approximately 1 g of cassava sample were weighed, and placed them in a 50 mL centrifuge tube containing 5 glass beads. 12.5 mL of pepsin–HCl solution (pepsin: 0.1 mol/L, pH = 1.0; HCl: 4 g/L) was added to each centrifuge tube. Then, tubes were capped, mixed on a vortex mixer, and incubated in a water bath (37 °C) for 30 min. Tubes were taken out of the water bath, 20 mL of sodium acetate buffer solution (0.1 mol/L) and 5 mL α-amylase were added to each sample, capped, vortexed, and immediately securely placed incubated at 39 °C in a shaking water bath for 0.25, 0.50, 0.75, 1, 2, 3, 4, 5, and 6 h. The shaking water bath was set at 160 strokes per min with a stroke length of 35 mm. Aliquots (0.5 mL) were pipetted from each tube at each incubation time, and added 20 mL ethanol (66%) to stop the digestion process. Next, centrifuge at 2200× g for 5 min to obtain the supernatant and determine the concentration of glucose according to the 3,5-dinitrosalicylic acid (DNS) method [32]. The total starch (TS) and free glucose (FG) contents in the solution were determined as described by Englyst et al. (1992) [10]. Starch digestion coefficient (DCt, %) and rapidly digestible starch (RDS), slowly digestible starch (SDS), digestible starch (DS), and resistant starch (RS) at different incubation times were calculated based on the following formula by Weurding et al. (2001 a) [31]:

DCt (%) = [(Gt − FG) × 0.9]/TS × 100(1)

RDS (%) = 1.16 × DC_2h_ − 21.49 (2)

SDS (%) = 1.29 × DC_4h_ − 30.86 − RDS (3)

DS (%) = RDS + SDS(4)

RS (%) = TS − DS(5)

where Gt is the content of glucose present at incubation time t. DC_2h_ and DC_4h_ is starch digestion coefficient at incubation time 2 h and 4 h, respectively. Moreover, 0.9 is the coefficient to convert molecular mass from glucose to starch monomer units.

Since in vitro starch digestion follows first-order kinetics, the following curve equation was used to estimate in vitro starch digestibility [31].

DC_t_ = DST × (1 − e^−KDS×t^) (6)

where DC_t_ (%) is the starch digestibility at incubation time t, and DST (%) is the content of digestible starch that will digest at a fractional rate of KDS (per unit time, h^−1^). The slope of each curve was calculated by Excel software, giving the KDS value.

### 2.2. In Vivo Study and Design

#### 2.2.1. Animals, Diets, and Management

The study utilized a 3 × 3 design, using processing methods (mechanical crushing (MC), steam conditioning (SC), and puffing conditioning (PU)) and conditioning temperatures (60, 75, and 90 °C) as main factors. A total of 1008 one-day-old male Cobb broilers (47.5 ± 2 g) purchased from a commercial hatchery (Dacheng Poultry Co., Ltd., Shanxi, China) were randomly divided into 9 treatments with 8 replicates (cages) (14 broilers per replicate). The broilers were fed a cassava–soybean basal diet with 0.4% titanium dioxide (TiO_2_) as an indigestible marker to determine nutrient digestibility. The composition of the starter (1–21 d of age) and grower (22–42 d of age) periods was formulated with reference to the recommended according to the NRC (1994) [33] to meet the nutrient requirements of broilers (Table 1). All feed ingredients were ground using a hammer mill (CM 100, Beijing Grinder Instrument Co., Ltd., Beijing, China) and then processed with the processing method of Section 2.1.1 to make pellet feed in Lintao Boya Co., Ltd. (Gansu, China). 60, 75, or 90 °C was the pelleting temperature of the diet. The temperature, lighting, and immunization program were according to the recommendation of the Cobb Broiler Management Guide [34]. Feed and water were offered ad libitum. The weighing was performed at a consistent time before morning feeding. Feed and residues per replicate provided by broilers were then weighed every three weeks for 42 d to determine average daily gain (ADG), average daily feed intake (ADFI), and feed-to-gain ratio (F/G). The mortality of broilers was recorded daily to correct the feed intake.

#### 2.2.2. Apparent Digestibility of Ileum and Total Tract

The apparent digestibility of the ileum and total tract were determined utilizing TiO_2_ by the marker ratio technique [35]. On days 21 and 42 of the study, four broilers were randomly selected from each replicate and slaughtered by cervical dislocation. The ileum was isolated between the Meckel diverticulum and the ileocecal junction, and digesta samples of all the broilers within each replicate were collected to homogenize, lyophilized, ground, and immediately frozen at −80 °C for analyzing dry matter (DM), crude protein (CP), and starch content to calculate the apparent ileum digestibility of nutrients.

At 18 to 21 d of age and 38 to 41 d of age, excreta was collected twice daily from each replicate (cage). Daily excreta from each cage was collected and mixed well before subsampling. Feather and dander were removed from the excreta and were acidified with 10% H_2_SO_4_ (*v*/*w* = 1:10). The excreta and feed samples were dried at 65 °C, ground into 1 mm screen with a hammer mill (CM 100, Beijing Grinder Instrument Co., Ltd., Beijing, China) for the determination of DM, CP, and apparent metabolizable energy (AME). The contents of DM (method 930.15) and CP (method 954.01) in feed, excreta, and ileum digesta samples were measured using standard procedures of AOAC (1995) [36]. TiO_2_ was determined according to the method of Short et al. (1996) [37]. Gross energy (GE) was measured with a calorimeter (C3000, IKA-WERKE Co., Ltd., Staufen, Germany). The AME (MJ/kg) was calculated according to the formula provided by Liu et al. (2020) [38] and Karunaratne et al. (2018) [39]. The following equations were used to calculate apparent nutrient digestibility and AME:

Apparent nutrient digestibility (%) = 100 − [(TiO_2_ in diet/TiO_2_ in excreta or ileum digesta) × (Nutrient in excreta or ileum digesta/nutrient in diet)] × 100 (7)

AME (MJ/kg diet) = [Feed intake × GE in diet] − (Excreta output × GE in excreta)]/Feed intake(8)

### 2.3. Statistical Analysis

The data were analyzed by two-way factorial arrangement (ANOVA) to determine the main effects (conditioning temperature and processing methods) and their interaction using the General Linear Models procedure of SPSS statistical software (Version 16.0 for Windows, SPSS Inc., Chicago, IL, USA). A cage served as an individual experimental unit. The Duncan method was used for multiple comparisons when the difference was significant. All results were presented as mean values and the standard error of the mean (SEM). The difference between means is significant (*) if *p* ≤ 0.05 and highly significant if *p* < 0.01 (**).

## 3. Results

### 3.1. In Vitro Digestibility of Cassava Starch

In vitro digestibility of cassava starch increased gradually with increased incubation time (0.25–3 h) until reaching an asymptotic level (Table 2). The starch digestibility reached the asymptotic level earlier when the conditioning temperature was 90 °C or PU. Starch digestibility at conditioned 90 °C was higher (*p* < 0.01) than at 60 °C or 75 °C, and PU demonstrated the highest (*p* < 0.01) digestibility, followed by SC and MC (0.25–2 h). The interactions (*p* < 0.05) were observed between processing methods and conditioning temperatures on starch digestibility (0.25–2 h) and starch digestion rate. The starch digestion rate varied from 2.7% (combination of 60 °C and MC) to 5.8% (combination of 75 °C and PU), with a difference of 3.1% between maximum and minimum values. The digestibility of cassava starch was highest at 60 °C or 75 °C in combination with PU.

### 3.2. In Vitro Digestion Properties of Cassava Starch

The amylose content and amylose/amylopectin at conditioned 60 °C were lower (*p* < 0.01) than at 75 °C or 90 °C, whereas the opposite was true for amylopectin content (*p* < 0.01) (Table 3). The RDS content at conditioned 90 °C was higher (*p* < 0.01) than at 60 °C or 75 °C, whereas the opposite was true for SDS content (*p* < 0.01). The amylose (24.8% vs. 26.7%), amylose/amylopectin (0.54 vs. 0.62), and SDS (2.4% vs. 6.5%) content of PU were lower (*p* < 0.01) than SC, whereas the opposite was true for amylopectin and RDS content (*p* < 0.01). The RS content of SC or PU was lower (*p* < 0.01) than MC. Significant interactions (*p* < 0.05) were observed between conditioning temperatures and processing methods on the content of amylose, amylopectin, amylose/amylopectin, RDS, SDS, and RS.

### 3.3. Growth Performance

Broilers fed diets conditioned at 90 °C had higher (*p* < 0.05) ADFI than those fed diets conditioned at 75°C or 60 °C in the starter period (Table 4). ADG and ADFI were the lowest (*p* < 0.05) for broilers fed MC diets in the starter period. In the grower period, broilers fed diets conditioned at 60 °C had higher (*p* < 0.05) ADG than those fed diets conditioned at 90 °C. Broilers fed PU diets had higher (*p* < 0.05) ADG and ADFI than those fed MC and SC diets. Broilers fed diets conditioned at 90 °C or PU diets had higher (*p* < 0.05) F/G in both the starter and grower periods. No interactions (*p* > 0.05) between processing methods and conditioning temperatures were observed for ADG, ADFI, and F/G in both the starter and grower periods.

### 3.4. Apparent Digestibility of Ileum

Broilers fed diets conditioned at 60 °C had higher (*p* < 0.05) ileum apparent digestibility of CP than those fed diets conditioned at 90 °C in both the starter and grower periods (Table 5). Broilers fed PU diets had higher (*p* < 0.01) ileum apparent digestibility of CP than those fed MC diets (76.0% vs. 72.3%) in the starter period. The ileum apparent digestibility of starch was higher (*p* < 0.05) for broilers fed SC diets than for those fed MC diets in both the starter and grower periods. Significant interactions (*p* < 0.05) were observed between processing methods and conditioning temperatures on CP apparent digestibility in the starter period and starch apparent digestibility in both the starter and grower periods. The combination of 60 °C and PU diets generated the highest ileum apparent digestibility of CP (78.1%) in the starter period (*p* < 0.05).

### 3.5. Apparent Digestibility of the Total Tract

The DM apparent digestibility of the total tract was not affected (*p* > 0.05) by processing methods and conditioning temperatures in both the starter and grower periods (Table 6). Broilers fed diets conditioned at 90 °C had lower (*p* < 0.05) AME than those fed diets conditioned at 75 °C or 60 °C in both the starter and grower periods. In the starter period, broilers fed PU diets had higher (*p* < 0.05) nitrogen retention than those fed MC or SC diets. However, broilers fed SC diets had higher (*p* < 0.05) AME than those fed MC diets in both the starter and grower periods. Significant interactions (*p* < 0.05) were observed between processing methods and conditioning temperatures on AME in the grower period.

## 4. Discussion

Since in vitro studies could be used to measure digestibility without sacrificing animals, many scholars have used the in vitro starch degradation method of the digestive tract model to simulate the in vivo hydrolysis of starch, and the in vitro time could be used to represent different parts of the small intestine [7,10,11,40]. It is generally believed that RDS refers to the starch that can be digested and absorbed in the small intestine within 20 min, and SDS refers to the starch that can be completely digested and absorbed in the small intestine within 20 to 120 min. RS cannot be digested and absorbed in the small intestine within 120 min but can reach the colon and be fermented by colonic microorganisms [10]. However, this definition applies to food nutrition but has limitations in animal nutrition. Weurding et al. (2001 b) [41] found that starch digestibility after 2 and 4 h incubation in vitro correlated well with starch digestibility in jejunum and ileum in broilers. Therefore, we adopted the regression equation proposed by Weurding et al. (2001 b) [41] and took the incubation for 2 and 4 h as the dividing points for the calculation of RDS, SDS, and RS.

For most native starches, the in vitro digestion profile reaches an asymptotic level after 6 h [31]. In the present study, the digestibility of cassava starch reaches asymptotic levels after 4 or 2 h when the conditioning temperature was 90 °C or PU, and the digestibility within 0.25–2 h was higher than other treatments, indicating that the increase of the conditioning temperature destroyed the hydrogen bonds between starch molecules, which increased the gelatinization degree of starch and promoted digestion of starch [28]. And PU also changes the physical and chemical structure of starch, increasing its digestibility during enzymatic hydrolysis [42]. Moreover, compared with MC, SC and PU, increased digestion rate and starch digestibility for 0.25–1 h, which is similar to the previous reports [22,40].

Starch digestion is mainly driven by amylase, a glycolytic enzyme that degrades starch into glucose, making it easier to absorb in the small intestine, and amylase activity is not only affected by external factors but also by the structure of starch itself (such as amylose, amylopectin, RDS, SDS, DS, and RS) [20,43]. The PU decreased the content of amylose, amylose/amylopectin, and SDS and increased the content of amylopectin and RDS, indicating that PU caused the starch structure to lose its integrity, the size of starch granules decreased, and the increase in the surface area led to an increase in its sensitivity to amylase, which promoted starch digestion [7,20].

The temperature required to promote starch digestion is not uniform but related to the characteristics of the starch varieties and processing methods. In vitro digestibility and digestion rate of cassava starch increase with increasing conditioning temperature, which is consistent with the findings obtained by Ali et al. (2020) [44]. Interestingly, MC had the lowest in vitro starch digestibility (0.25–2 h) and digestion rate at a conditioning temperature of 60 °C, while PU had the highest starch digestibility (0.5–1 h) at a conditioning temperature of 75 °C. Ali et al. (2020) [44] reported that potato starch was PU at a conditioning temperature of 100 °C had the lowest in vitro starch digestibility, while corn starch was PU at 160 °C had the highest digestibility. This may be due to the fact that no moisture was added during the PU process in this study. If in the presence of moisture, high temperature leads to high gelatinization of starch, thereby increasing the sensitivity of starch to enzymatic hydrolysis [44]. Compared with SC, the RS content of wheat was similar at 20, 60, and 75 °C conditioning temperatures after MC, and the RS content was the highest at 90 °C [45]. However, in our study, SC and PU had the lowest RS content at the conditioning temperature of 75 °C, not 90 °C. Moreover, cassava starch had lower amylose content and amylose/amylopectin at a conditioning temperature of 60 °C in combination with PU or SC. It shows that different types of starch use different processing methods, and the optimum temperature for promoting starch digestion is different. Furthermore, the amylose content, amylose/amylopectin, and RDS content of cassava at conditioned 60 °C were lower than 75 °C or 90 °C, whereas the amylopectin and RS content were the highest, which is in agreement with the results of Wang et al. (2019) [27], who found the conditioning temperature increased from 65 °C to 85 °C and the RS content in sorghum increased. This may be because amylopectin was more sensitive to high temperature than amylose, and many of its branch points were easily broken, which led to the formation of linear fragments similar to amylose [7]. Additionally, high temperature leads to high gelatinization of starch, increasing the susceptibility of starch to enzymatic hydrolysis [44]. The gelatinization of starch has a direct impact on the digestibility of starch, as in which crystal structure is changed and the glycosidic bonds are destroyed, and therefore the molecules become easily accessible to digestive enzymes, the content of amylopectin is increased, and the amylose/amylopectin was decreased, thereby leading to increased digestibility [20,44].

Broilers fed PU diets had higher ADFI than those fed MC diets. Broilers fed diets conditioned at 90 °C or PU diets had higher F/G in both the starter and grower periods. These results agree with those of Liermann et al. (2019) [46], who demonstrated that broilers fed PU diets had higher ADG, final weight (day 35), and ADFI compared to those fed MC diets. In fact, the puffing cassava starch granules were disintegrated into small molecular substances that were easier to digest and absorb, and the viscosity was reduced, the structure was loose, the volume was increased, and the protein was denatured and more easily degraded by proteases [42]. Moreover, the denatured starch and protein reduced the stress of the broiler intestinal flora and promoted the development of the digestive system and the establishment of the intestinal micro-ecosystem [47], which further affects the growth performance of broilers. Broilers fed SC diets had higher ADG and lower F/G compared to MC diets; this finding is in line with the concluding remarks of Naderinejad et al. (2016) [48], who reported that broilers fed MC diets had lower FI and body weight gain than those fed SC diets. Furthermore, compared with MC corn diets, SC diets increased ADG and in vitro, true digestibility, and decreased F/G in yaks [40]. In our study, the PU diets had the highest ADFI and F/G, indicating that the PU diets were more palatable. Although the SC diets did not increase ADFI, it had the lowest F/G, the highest AME, and relatively high ileum starch digestibility, indicating that the rapid digestion of starch in the ileum is conducive to improving the AME and feed conversion rate of broilers, thereby promoting growth performance [31].

Broilers fed diets conditioned at 90 °C had higher ADFI than those fed diets conditioned at 75 or 60 °C in the starter period. In the grower period, broilers fed diets conditioned at 60 °C had higher ADG than those fed diets conditioned at 90 °C, which is consistent with the findings of Abdollahi et al. (2011) [45], who found that the growth performance (consumed more feed) of broilers decreased with increased conditioning temperature. The F/G of broilers increased with the increase in conditioning temperature. In addition, Abdollahi et al. (2020) [49] also found that broilers fed diets conditioned at 90 °C had higher F/G and consumed more feed than the unconditioned diets, but similar to the 60 °C conditioned diets. A study on nursery pigs also found that the F/G of high-temperature conditioned (88 °C) diets was higher than low temperature (54 °C) diets [24]. A possible explanation was that high-temperature conditioning could make starch gelatinization and anti-nutritional factors degrade, thereby improving the performance of nutrients and improving the nutritional value of poultry diets, but high-temperature conditioning would also inactivate enzymes and vitamins in the diet and reduced the utilization of protein and starch [49].

It is worth noting that the effective utilization of starch is not only related to the digestion rate but also depends on the passage time of starch in the digestive tract; the longer the starch stays in the small intestine, the more complete the starch digestion [50]. Broilers fed PU diets had higher starch apparent digestibility of ileum in the starter and grower periods than those fed MC diets. There was also an increase in starch digestibility of ileum if corn, wheat, and sorghum diets were PU [51]. This increase is likely the result of starch gelatinization, which breaks down the intermolecular bonds in the starch granules so that the gelatinized starch is more readily available to intestinal enzymes [52]. The puffing increases RDS content in barley diets, peas diets, or diets containing potato starch and wheat bran, reducing RS content and thus increasing ileum starch digestibility in pigs [53]. In our study, the benefits observed in RDS, amylopectin, and RS were also caused by starch gelatinization. Broilers fed PU diets had higher ileum digestibility of CP and nitrogen retention in the starter period than those fed MC diets. However, the PU had no effect on the apparent digestibility of DM in the total tract and the nitrogen retention in the grower period, which indicated that the puffing promoted the digestion of nutrients in the small intestine. It may be that the heat generated by puffing changes the three-dimensional structure of the protein, thereby increasing the contact of the digestive enzymes with the peptide bonds [54]. Additionally, Rodriguez et al. (2020) [51] found that puffing corn increased the standardized ileum digestibility of CP and all amino acids except lysine and proline, but CP and amino acids digestibility in wheat and sorghum diets were not affected by PU. This may be related to the types of proteins present in corn, wheat, and sorghum, which differ in their susceptibility to puffing [51].

The AME was lower for MC diets than for SC and PU diets during both the starter and grower periods. Rodriguez et al. (2020) [51] reported that PU improved the total tract apparent digestibility of energy from corn diets and sorghum diets in finishing pigs but failed to improve the metabolizable energy of wheat diets in pigs. Another study found that although feed particle size had no effect on broiler AME, SC diets (70 °C) increased the apparent digestibility of starch in the ileum [48]. The increased AME of PU may be a consequence of the increased apparent ileum digestibility of starch and CP that we observed, but more studies are needed to verify this hypothesis.

High temperature during the puffing process leads to the denaturation of proteins or inhibits the action of digestive enzymes. Meanwhile, an increased conditioning temperature increases water-soluble non-starch polysaccharides in the intestine, resulting in an increased viscosity of the digesta and influencing the digestion of starch [55]. Therefore, the digestibility of nutrients varies with the conditioning temperature of the diets. Our study found that the ileum digestibility of DM and starch in the diets was not influenced by the conditioning temperature. Nevertheless, the CP digestibility of diets conditioned at 60 °C was higher than that conditioned at 75 °C or 90 °C. This result is different from the results of Wang et al. (2019) [27], who evaluated the influence of the different conditioning temperatures (65, 70, 75, 80, and 85 °C) of sorghum-based diets on nutrition digestibility in pigs and observed that the ileum digestibility of starch and CP was higher at 75 and 80 °C than at other temperatures. Lundblad et al. (2012) [56] found that the ileum starch digestibility of wheat diets increased with increasing conditioning temperature but had no effect on DM and CP digestibility. On the one hand, during the conditioning process, high temperature and interaction between water and sugar lead to the Maillard reaction, inhibiting starch gelatinization and thereby reducing nutrient absorption [44,46]. On the other hand, high temperature caused the formation of a disulfide oligomer complex between sulfur-containing amino acids, resulting in a change of the protein’s secondary structure.

The conditioning temperature of SC diets at either 60 °C or 90 °C decreased the ileum digestibility of DM, compared with those fed nonconditioned MC diets. Moreover, broilers fed diets conditioned at 60 °C had higher ileum digestibility of DM and starch compared to those conditioned at 90 °C [49]. Similarly, Abdollahi et al. (2011) [45] compared the wheat mash diets of unconditioned MC or SC at 60, 75, or 90 °C and reported that SC resulted in slightly improved N and starch digestibility at 60 °C. However, increasing conditioning temperature from 60 to 75 and 90 °C both decreased N and starch digestibility. These results may at least partly explain the reduced digestibility of diets starch under high temperature conditions. This was inconsistent with our results that the ileum digestibility of starch in broilers fed SC diets was higher than in MC diets regardless of conditioning temperature, which may also result in higher ADG and lower F/G in SC diets than in MC diets, part of the reason. The PU diets had the highest AME at a conditioning temperature of 60 °C and the lowest at 90 °C. This is similar to the results of Abdollahi et al. (2011) [45] that increasing the conditioning temperature from 60 to 90 °C with SC diets decreased the AME of the diets. Once again, it is clear that the processing methods and conditioning temperatures had an effect on the biochemical properties of the diets, which leaded to a change in digestibility. Therefore, the potential nutritional value of diets could be improved through an effective combination of conditioning temperatures and processing methods.

## 5. Conclusions

In conclusion, cassava starch promoted starch digestion rate by reducing amylose content and amylose/amylose under PU combined with a conditioning temperature of 60 °C. The ileum digestibility of starch in broilers fed SC diets was higher than MC diets regardless of conditioning temperatures. And SC diets increased AME and decreased F/G to promote growth performance of broilers. In addition, high conditioning temperature is not conducive to poultry feed manufacture. The current findings may suggest that broiler diets can be conditioned at lower temperatures if combined with puffing or steam conditioning and also benefit from improved feed utilization.

## Figures and Tables

**Table 1:** Ingredients compositions of the experimental diets in starter and grower periods (as-fed-basis).

Ingredient (%)	Starter (1–21 d)	Grower (22–42 d)
Cassava	55.0	57.0
Soybean flour	30.04	25.26
Vegetable oil	4.00	6.00
Corn gluten flour	3.00	4.00
Fish meal	4.00	4.00
CaHP0_4_	1.45	1.05
Limestone	0.70	0.60
Premix ^1^	0.50	1.00
Sodium chloride	0.41	0.41
DL-methionine	0.25	0.13
Lysine	0.25	0.15
Titanium dioxide	0.40	0.40
Total	100.0	100.0
Nutrient levels (%) ^2^		
ME, MJ/kg ^3^	12.34	12.97
Crude protein	20.0	18.0
Calcium	1.00	0.90
Available phosphorous	0.45	0.38
Methionine	0.54	0.41
Lysine	1.25	1.05
Threonine	0.82	0.73
Tryptophan	0.21	0.19
Arginine	1.21	1.12
Leucine	1.25	1.05
Histidine	0.35	0.32
Valine	0.84	0.73

^1^ Provided per kilogram of diets: vitamin A (as all-trans-retinol), 12,000 IU; cholecalciferol, 3500 IU; Vitamin E (as d-α-tocopherol), 44.7 IU; vitamin B_12_, 0.2 mg; biotin, 0.1 mg; niacin, 50 mg; vitamin K_3_, 2 mg; pantothenic acid, 12mg; folic acid, 2 mg; thiamine, 2 mg; riboflavin, 6 mg; pyridoxine hydrochloride, 5 mg; Mn, 80 mg; Fe, 60 mg; Cu, 8 mg; I, 1 mg; Co, 0.3 mg. ^2^ Nutrient levels were calculated using values from the China Feed Database. ^3^ ME, metabolizable energy.

**Table 2:** Effect of processing methods and conditioning temperatures on in vitro digestibility of cassava starch at different incubation times and starch digestion rates.

Items		Incubation Time (h)									SDR (h^−1^)
		0.25	0.50	0.75	1	2	3	4	5	6	
MC	60 °C	62.4 ^d^	73.5 ^g^	78.0 ^e^	82.0 ^f^	89.3 ^d^	97.0	97.9	98.3	98.0	2.70 ^f^
	75 °C	67.1 ^c^	80.9 ^e^	84.5 ^d^	88.5 ^de^	92.9 ^b^	98.2	98.2	97.6	98.1	3.39 ^e^
	90 °C	73.6 ^b^	83.9 ^d^	89.9 ^c^	91.2 ^d^	97.2 ^a^	98.0	98.2	98.5	98.5	4.05 ^d^
SC	60 °C	69.0 ^c^	80.9 ^e^	83.6 ^d^	88.7 ^de^	91.9 ^bc^	97.3	98.2	98.4	98.6	3.33 ^e^
	75 °C	69.1 ^c^	78.2 ^f^	83.0 ^d^	87.1 ^e^	90.6 ^cd^	98.1	98.7	98.5	98.0	3.21 ^e^
	90 °C	75.8 ^b^	87.2 ^c^	92.3 ^ab^	95.1 ^ab^	97.4 ^a^	97.6	98.3	97.9	98.6	4.57 ^c^
PU	60 °C	85.1 ^a^	91.7 ^ab^	93.9 ^a^	97.8 ^a^	98.5 ^a^	98.2	98.0	98.6	98.2	5.74 ^a^
	75 °C	82.9 ^a^	93.4 ^a^	94.3 ^a^	98.3 ^a^	97.9 ^a^	97.9	98.8	98.4	98.1	5.81 ^a^
	90 °C	81.5 ^a^	90.0 ^b^	91.6 ^bc^	92.5 ^bc^	98.2 ^a^	98.1	98.1	98.6	98.0	4.91 ^b^
SEM ^1^		1.45	1.07	0.98	1.26	0.68	0.46	0.71	0.84	0.92	0.07
Main effect											
Conditioning temperatures											
60 °C		72.2	82.0	85.2	89.5	93.2	97.5	98.0	98.4	98.3	3.92
75 °C		72.9	84.1	87.3	91.3	93.9	98.1	98.6	98.2	98.1	4.13
90 °C		77.3	87.0	91.3	92.9	97.6	97.9	98.2	98.3	98.4	4.50
Processing methods											
MC		67.7	79.4	84.1	87.2	93.1	97.8	98.1	98.1	98.2	3.38
SC		71.2	82.1	86.3	90.2	93.3	97.7	98.4	98.3	98.4	3.71
PU		83.5	91.7	93.3	96.3	98.2	98.1	98.3	98.5	98.1	5.48
*p*-value											
Conditioning temperatures		**	**	**	**	**	NS	NS	NS	NS	**
Processing methods		**	**	**	**	**	NS	NS	NS	NS	**
Conditioning temperatures × Processing methods		**	*	*	*	**	NS	NS	NS	NS	**

^a–g^ Mean values without common superscripts within columns differ significantly. NS, not significant, *p* > 0.05; * *p* ≤ 0.05, ** *p* ≤ 0.01. ^1^ SEM, standard error of means. MC, mechanical crushing; SC, steam conditioning; PU, puffing conditioning; SDR, starch digestion rate.

**Table 3:** Effect of processing methods and conditioning temperatures on total starch, amylose, amylopectin, rapidly digestible starch, slowly digestible starch, digestible starch, and resistant starch content (%).

Items		TS	Amylose	Amylopectin	Amylose/amylopectin	RDS	SDS	DS	RS
MC	60 °C	71.3	21.6 ^d^	49.7 ^a^	0.43 ^d^	58.5 ^b^	9.5 ^a^	68.0	3.28 ^a^
	75 °C	70.0	27.0 ^b^	42.9 ^cde^	0.63 ^b^	60.4 ^b^	6.7 ^c^	67.1	3.02 ^cd^
	90 °C	70.3	28.9 ^a^	41.4 ^e^	0.61 ^a^	64.1 ^a^	3.2 ^d^	67.3	3.02 ^cd^
SC	60 °C	70.2	24.6 ^c^	45.7 ^bcd^	0.54 ^c^	59.7 ^b^	7.5 ^bc^	67.3	3.01 ^cd^
	75 °C	70.9	29.0 ^a^	41.9 ^de^	0.65 ^a^	59.3 ^b^	9.0 ^ab^	68.4	2.62 ^e^
	90 °C	70.3	26.7 ^b^	43.5 ^cde^	0.61 ^b^	64.3 ^a^	3.1 ^d^	67.4	2.90 ^d^
PU	60 °C	70.6	24.5 ^c^	46.2 ^abc^	0.53 ^c^	65.6 ^a^	1.9 ^d^	67.5	3.22 ^ab^
	75 °C	70.4	26.8 ^b^	44.6 ^bcde^	0.60 ^b^	65.7 ^a^	3.2 ^d^	68.9	2.50 ^e^
	90 °C	70.4	22.5 ^d^	47.8 ^ab^	0.47 ^d^	65.1 ^a^	2.2 ^d^	67.8	3.09 ^bc^
SEM ^1^		0.96	2.13	1.42	0.02	0.46	0.35	0.89	0.09
Main effect									
Conditioning temperatures									
60 °C		70.7	23.5	47.2	0.50	61.2	6.3	67.6	3.1
75 °C		70.8	27.3	43.1	0.64	61.0	6.3	68.1	2.7
90 °C		70.3	26.8	44.3	0.59	64.5	2.8	67.4	3.0
Processing methods									
MC		70.5	25.8	44.7	0.59	61.0	6.4	67.5	3.1
SC		70.5	26.7	43.8	0.62	61.3	6.5	67.7	2.8
PU		70.8	24.8	46.2	0.54	65.5	2.4	67.9	2.9
*p*-value									
Conditioning temperatures		NS	**	**	**	**	**	NS	**
Processing methods		NS	**	**	**	**	**	NS	**
Conditioning temperatures × Processing methods		NS	*	*	*	**	**	NS	*

^a–e^ Mean values without common superscripts within columns differ significantly. NS, not significant, *p* > 0.05; * *p* ≤ 0.05, ** *p* ≤ 0.01. ^1^ SEM, standard error of means. MC, mechanical crushing; SC, steam conditioning; PU, puffing conditioning; TS, total starch; RDS, rapidly digestible starch; SDS, slowly digestible starch; DS, digestible starch; RS, resistant starch.

**Table 4:** Effect of processing methods and conditioning temperatures on performance parameters of broilers.

Items		Starter (1–21 d)			Grower (22–42 d)		
		ADG (g)	ADFI (g)	F/G	ADG (g)	ADFI (g)	F/G
MC	60 °C	37.3	54.7	1.47	85.3	188	2.21
	75 °C	36.6	56.3	1.54	82.1	187	2.27
	90 °C	37.1	58.1	1.57	85.0	192	2.26
SC	60 °C	38.9	55.1	1.42	86.0	185	2.15
	75 °C	38.1	55.7	1.46	85.8	182	2.12
	90 °C	37.6	58.9	1.57	83.0	192	2.31
PU	60 °C	38.6	57.9	1.50	89.3	203	2.27
	75 °C	37.9	57.6	1.52	87.2	204	2.34
	90 °C	36.8	57.6	1.56	86.1	207	2.40
SEM ^1^		0.23	0.81	0.04	1.35	2.42	0.03
Main effect							
Conditioning temperatures							
60 °C		38.3	55.9 ^b^	1.46 ^c^	86.9 ^a^	192	2.21 ^b^
75 °C		37.5	56.5 ^b^	1.51 ^b^	85.0 ^ab^	191	2.25 ^b^
90 °C		37.2	58.2 ^a^	1.57 ^a^	84.7 ^b^	197	2.32 ^a^
Processing methods							
MC		37.0 ^b^	56.4 ^b^	1.52 ^a^	84.1 ^b^	189 ^b^	2.25 ^b^
SC		38.2 ^a^	56.6 ^b^	1.48 ^b^	84.9 ^b^	186 ^b^	2.19 ^c^
PU		37.8 ^ab^	57.7 ^a^	1.53 ^a^	87.5 ^a^	205 ^a^	2.34 ^a^
*p*-value							
Conditioning temperatures		NS	*	*	*	NS	**
Processing methods		*	*	*	**	*	**
Conditioning temperatures × Processing methods		NS	NS	NS	NS	NS	NS

^a–c^ Mean values without common superscripts within columns differ significantly. NS, not significant, *p* > 0.05; * *p* ≤ 0.05, ** *p* ≤ 0.01. ^1^ SEM, standard error of means. MC, mechanical crushing; SC, steam conditioning; PU, puffing conditioning. ADG, average daily gain (g/broiler/day); ADFI, average daily feed intake (g/broiler/day); F/G, feed-to-gain ratio.

**Table 5:** Effect of processing methods and conditioning temperatures on ileum apparent nutrients digestibility of broilers.

Items		Starter (d 21, %)			Grower (d 42, %)		
		DM	CP	Starch	DM	CP	Starch
MC	60 °C	68.2	73.4 ^bc^	94.4 ^c^	75.9	76.9	95.1 ^bc^
	75 °C	71.1	72.2 ^c^	93.3 ^c^	74.6	75.2	94.2 ^c^
	90 °C	72.3	71.3 ^c^	95.9 ^bc^	80.7	73.1	96.4 ^ab^
SC	60 °C	71.2	76.2 ^ab^	97.6 ^a^	80.5	76.7	97.3 ^a^
	75 °C	72.1	73.8 ^bc^	97.9 ^a^	75.7	74.1	97.2 ^a^
	90 °C	72.9	74.5 ^b^	97.9 ^a^	76.0	73.3	97.0 ^a^
PU	60 °C	71.6	78.1 ^a^	96.6 ^b^	76.9	78.1	97.2 ^a^
	75 °C	73.8	76.2 ^ab^	96.8 ^b^	75.1	76.4	97.1 ^a^
	90 °C	69.2	73.6 ^bc^	96.2 ^b^	74.3	75.9	97.4 ^a^
SEM ^1^		0.57	0.76	0.22	0.66	0.67	0.38
Main effect							
Conditioning temperatures							
60 °C		70.3	75.9	96.2	77.7	77.2 ^a^	96.5
75 °C		72.3	74.1	95.9	75.1	75.2 ^b^	96.1
90 °C		71.4	73.1	96.6	77.0	74.1 ^b^	96.9
Processing methods							
MC		70.5	72.3	94.5	77.0	75.1	95.2
SC		72.0	74.8	97.7	77.3	74.7	97.1
PU		71.5	76.0	96.5	75.4	76.8	97.2
*p*-value							
Conditioning temperatures		NS	*	NS	NS	*	NS
Processing methods		NS	**	*	NS	NS	*
Conditioning temperatures × Processing methods		NS	*	*	NS	NS	*

^a–c^ Mean values without common superscripts within columns differ significantly. NS, not significant, *p* > 0.05; * *p* ≤ 0.05; ** *p* < 0.01. ^1^ SEM, standard error of means. MC, mechanical crushing; SC, steam conditioning; PU, puffing conditioning. DM, dry matter; CP, crude protein.

**Table 6:** Effect of processing methods and conditioning temperatures on total tract apparent nutrients digestibility and apparent metabolizable energy (AME) of broilers.

Items		Starter (18–21 d)			Grower (38–41 d)		
		DM (%)	Nitrogen Retention (%)	AME (MJ/Kg)	DM (%)	Nitrogen Retention (%)	AME (MJ/Kg)
MC	60 °C	71.2	65.2	13.01	71.6	67.2	13.34 ^b^
	75 °C	75.2	68.2	13.03	72.5	69.7	13.35 ^b^
	90 °C	73.9	68.8	12.94	72.1	74.9	13.28 ^bc^
SC	60 °C	75.6	69.2	13.22	73.4	71.2	13.37 ^b^
	75 °C	74.9	66.8	13.16	71.3	69.7	13.51 ^ab^
	90 °C	76.3	69.5	12.92	72.4	68.7	13.42 ^b^
PU	60 °C	75.3	73.6	13.13	73.3	74.4	13.65 ^a^
	75 °C	73.4	76.2	13.06	70.8	66.1	13.28 ^bc^
	90 °C	73.0	75.6	13.05	69.5	65.9	13.23 ^c^
SEM ^1^		0.65	1.24	0.26	0.35	1.17	0.50
Main effect							
Conditioning temperatures							
60 °C		74.1	69.27	13.12 ^a^	72.80	70.96	13.45
75 °C		74.5	70.40	13.08 ^a^	71.56	68.54	13.38
90 °C		74.4	71.30	12.97 ^b^	71.35	69.86	13.31
Processing methods							
MC		73.4	67.33 ^b^	12.99 ^b^	72.10	70.62	13.32
SC		75.6	68.50 ^b^	13.10 ^a^	72.39	69.88	13.43
PU		73.9	75.13 ^a^	13.08 ^a^	71.23	68.88	13.39
*p*-value							
Conditioning temperatures		NS	NS	*	NS	NS	*
Processing methods		NS	*	*	NS	NS	*
Conditioning temperatures × processing methods		NS	NS	NS	NS	NS	*

^a–c^ Mean values without common superscripts within columns differ significantly. Not significant (NS), *p* > 0.05; * *p* ≤ 0.05. ^1^ SEM, standard error of means. MC, mechanical crushing; SC, steam conditioning; PU, puffing conditioning; DM, dry matter; AME, apparent metabolizable energy.

## Data Availability

Data sharing is not applicable.
